# Tumor resident microbiota and response to therapies: An insight on tissue bacterial microbiota

**DOI:** 10.3389/fcell.2022.1048360

**Published:** 2023-01-04

**Authors:** Francesca Pirini, Michela Cortesi, Maria Maddalena Tumedei, Michele Zanoni, Sara Ravaioli, Sara Bravaccini

**Affiliations:** IRCCS Istituto Romagnolo per lo Studio dei Tumori (IRST) “Dino Amadori”, Meldola, Italy

**Keywords:** tissue microbiota, response to therapies, microbiota manipulation, tumor microenvironment, precision medicine

## Abstract

The role of the intestinal microbiota in the promotion, progression, and response to therapies is gaining importance, but recent studies confirm the presence of microbiota also in the tumor, thus becoming a component of the tumor microenvironment. There is not much knowledge on the characteristics and mechanisms of action of the tumor resident microbiota, but there are already indications of its involvement in conditioning the response to therapies. In this review, we discuss recent publications on the interaction between microbiota and anticancer treatments, mechanisms of resistance and possible strategies for manipulating the microbiota that could improve treatments in a personalized medicine perspective.

## Introduction

Despite all the efforts to produce new therapies and identify predictive biomarkers of response to anti-cancer drugs, there is a percentage of patients with the same tumor biological characteristics and biomarker positivity who do not respond to therapies. Microbiota have been linked with cancer promotion and progression in different cancer types and recent studies revealed that gut microbiota can influence the response to current anti-cancer therapies (Alexander et al., 2017; Sepich-Poore et al., 2021; Shiao et al., 2021; Wong et al., 2021) thanks to a continuous cross-talk between the gut microbiota and various organs such as the lungs (Zhang et al., 2020), breast (Al-Ansari et al., 2021), pancreas (Kurashima et al., 2021), and brain (Javed et al., 2020). Through these axes, the intestinal microbiota is able to send metabolites, proteins and nucleic acids to the body districts, influencing the tumor pathogenesis and the response to treatment. However, the presence of the microbiota is not limited to the gut. In fact, microbes can reach the different body sites principally by passing through the barriers of the intestine and entering the lymphatic system or blood circulation, or through openings to the external environment such as the mammary ducts. Other microorganisms have unique methods to reach the tumor site. For instance, *Listeria* is able to infect antigen-presenting cells, including macrophages, dendritic cells and myeloid-derived suppressor cells (MDSCs), that can colonize the Tumor Microenvironment (TME), thus preventing its elimination from the immune response (Chandra et al., 2013; Quispe-Tintaya et al., 2013). Tumor resident microbiota is emerging as an integral component of the TME of different cancer types such as breast, lung and pancreatic cancer (Urbaniak et al., 2016; Riquelme et al., 2019; Nejman et al., 2020). Thanks to the application of next-generation sequencing (NGS), it was possible to identify the presence of nucleic acids of viruses, bacteria, fungi and protozoa. This allowed characterizing the resident microbiota from a phylogenetic point of view, confirming the presence of dysbiosis also at the tissue level in case of pathology and find a relationship between certain tissue microbiota profiles and the carcinogenesis in melanoma, glioblastoma and breast, lung, ovary, pancreas, bone cancers (Banerjee et al., 2018; Nejman et al., 2020). Later on, it was understood that some bacteria, like *Streptococcus gallolyticus*, need direct contact with the cell and to be in an exponential growth phase to condition cellular mechanisms and induce tumorigenesis in colon cancer (Kumar et al., 2017). Recently, Nejman and others (2020) showed the presence of bacteria inside tumor and immune cells in different tumor samples rather than in the extracellular space, observation also confirmed by Transmission Electron Microscopy (TEM). A very recent study conducted by Aikun Fu and others (2022) confirmed the presence of bacteria in tumor tissue, and their abundance mainly inside the cells. In this case, a spontaneous murine BT MMTV-PyMT model of breast cancer was used. High-resolution electron microscopy analysis confirmed a higher presence of bacteria-like structures in cytosol than in extracellular space. Confirmation of the intracellular localization of the bacteria could change the perspectives on the nature of the interaction between microbiota and cancer, unbalancing it mainly on the collaboration in favour of the tumor. These findings lead to a greater attention in the choice of therapies; for example, the intracellular localization of the bacteria make them difficult to get rid of with common antibiotics not able to cross the cell wall. Moreover, intracellular bacteria may be able to metabolize drugs and induce resistance. Another argument of discussion is whether the tissue microbiota and the gut microbiota have different roles in cancer pathogenesis and in response to therapies. At the moment there are various indications on the involvement of the tissue microbiota in cancer risk (Xuan et al., 2014), prognosis (Riquelme et al., 2019), and response to therapies (Geller et al., 2017), but in particular Aikun Fu’s study (2022) shows that the gut and tissue microbiota can play different roles at different times of carcinogenesis. The study carried out on a mouse model of breast cancer, suggests that the microbiota of the gut plays a role mainly in tumor growth, while the tissue microbiota is involved in metastases formation. However, it is necessary to consider that different tumors have different clinical characteristics and different microbiota profiles, therefore what has been reported above cannot be considered the rule. In this review, we aim to shed light on the current knowledge regarding the functional role of the tissue microbiota, focusing on its influence in the therapies response. We discuss the most recent papers and reviews on the association between therapy resistance and tissue microbiota. The literature search was performed using PubMed and web of science in particular using “local microbiome”, “tissue microbiome”, “locally resident microbiome”, and “treatment resistance” or “therapy response” or “pharmacomicrobiomics” as key words for the search. Only results on bacteria are reported.

## Local microbiome-mediated influence on cancer therapies and resistance mechanisms

Despite the innovations in the pharmacological field and the commitment to understand the mechanisms underlying drug resistance, the problem of inter-individual disparity in drug responses has not been solved yet. The microbiota, with more than 3,000 species of bacteria, viruses and fungi, is capable of producing millions of metabolites and is equipped with millions of protein-coding genes. Therefore, it is likely involved in pharmacokinetics (Zhang J. et al., 2018). Recent evidence showed the relationship between the gut microbiota and anticancer treatments, such as chemotherapy (Alexander et al., 2017), radiotherapy (Shiao et al., 2021), targeted therapy (Wong et al., 2021), and immunotherapy (Sepich-Poore et al., 2021). However, we must not forget that most of the therapeutic nucleotides are activated inside the cells and that the bacteria at the local level are mainly intracellular. The study of the tissue microbiota is very recent but clear evidence of its contribution to pharmacokinetics and resistance has already been reported. Based on literature, three main possible mechanisms have been reported by which the tissue microbiota can influence therapies: i) the biotransformation of drugs by bacterial enzymes; ii) the immune reprogramming, iii) the alteration of important cellular biological processes like apoptosis, cell cycle and DNA repair.

### Biotransformation by bacterial enzymes

The bacteria have the ability to transform organic molecules, including drugs, by their unique enzimolome. Some microorganisms produce nucleoside analog-catabolizing enzymes that can interfere with nucleoside analogues (NAs) treatments. After internalization in the cell, NAs are activated through phosphorylation by enzymes and interfere with cellular nucleo(s) (t) ide metabolism and the synthesis of DNA/RNA. Therefore, NAs efficacy is dependent on the expression and activity of nucleo(s) (t) ide-metabolizing enzymes. In *mycoplasma* infected cell cultures has been reported a decrease of 10 to 140 fold of the biological activity of 5-FdUrd, 5-trifluorothymidine (TFT) and other halogenated dThd analogues due to the expression of pyrimidine nucleoside phosphorylase (PyNP) (Vande Voorde et al., 2012) that phosphorylate the drugs and dramatically compromised the cytostatic activity of various pyrimidine-based NAs. In order to elucidate the bacteria drug interaction, Lehouritis et al. (2015) tested the effect of two bacteria previously identified in breast tumor tissue, *Escherichia coli* and *Listeria welshimeri*, on the efficacy of 30 chemotherapeutic drugs used to treat cancer. The *in vitro* and *in vivo* experiments showed that the bacteria could both increase or decrease the cytotoxicity of drugs. For example, at the concentrations tested, the cytotoxicity of cladribine, gentamicin and anticancer antibiotics was decreased by bacteria, while fludarabine and 6-mercaptopurine-2-deoxyadenosine were activated. The HPLC and mass spectrometry analysis reveal that these effects can be due to the biotransformation of the drugs by the bacterial enzymes, as shown from the analysis of the gemcitabine and *E.coli* products. These findings were confirmed by Geller (2017). Geller observed that colorectal and pancreatic cancer cell lines co-cultured with human dermal fibroblasts (HDFs) were more resistant to gemcitabine. Later they found out that HDFs were contaminated by *Mycoplasma hyorhinis,* which is able to decrease the sensitivity of cancer cells to gemcitabine, as demonstrated *in vitro* and *in vivo,* by metabolization of gemcitabine in an inactive metabolite. They also tested 27 bacterial species and identified 13 other species with the same ability to confer resistance to gemcitabine. The resistance is due to the long isoform of the enzyme cytidine deaminase (CDD) that metabolizes gemcitabine into inactive metabolite 2′, 2′-difluorodeoxyuridine.

### Immune reprogramming

Intratumoral bacteria can affect the immune response and shape the tumor microenvironment, which in turn modulate the response to immunotherapy. A study by Pushalkar et al. (2018) on a KC and KPC mice model demonstrated that bacteria from gut can migrate to the pancreas and induce immune reprogramming, probably by the activation of Toll-like receptors in the TME. They also find that the removal of the tumor tissue bacteria in pancreatic cancer induces immunogenic reprogramming of the tumor microenvironment and increases PD-1 expression on CD4^+^ CD8+T cells. Therefore, the treatment of PDA patients with antibiotics can be a possible strategy to increase the efficacy of immunotherapy. The activation of TLR4 and MYD88 innate immune signalling by *F. nucleatum* has been observed in *in vitro* co-culture studies, as well as xenograft-based nude BALB/c mouse models and it altered the response to 5-fluorouracil and oxaliplatin chemotherapies as well as miR-18 and miR-4802, presumably activating the autophagy pathway (Yu et al., 2016). A study by Kalaora et al. (2021) identified antigens derived from bacteria on tumor HLA-I and HLA-II molecules both on tumor cells and antigen presenting cells in melanoma tumors samples and metastasis from different patients. These findings leads to the possibility that intratumoral bacteria may affect T cell immune reactivity and modulate immune function. The type of bacteria and them localization should be taken into account for the selection of immunotherapies. The microbiota effect can be also positive. For example, in mice the translocation of Gram-positive bacteria into mesenteric lymph nodes and spleen can increase the response to Cyclophosphamide (CTX) due to the stimulation of Th1 and Th17 immune response. In contrast, animals treated with antibiotics developed a resistance to the treatment (Viaud et al., 2013; Daillère et al., 2016).

### Other mechanisms

**FIGURE 1:**
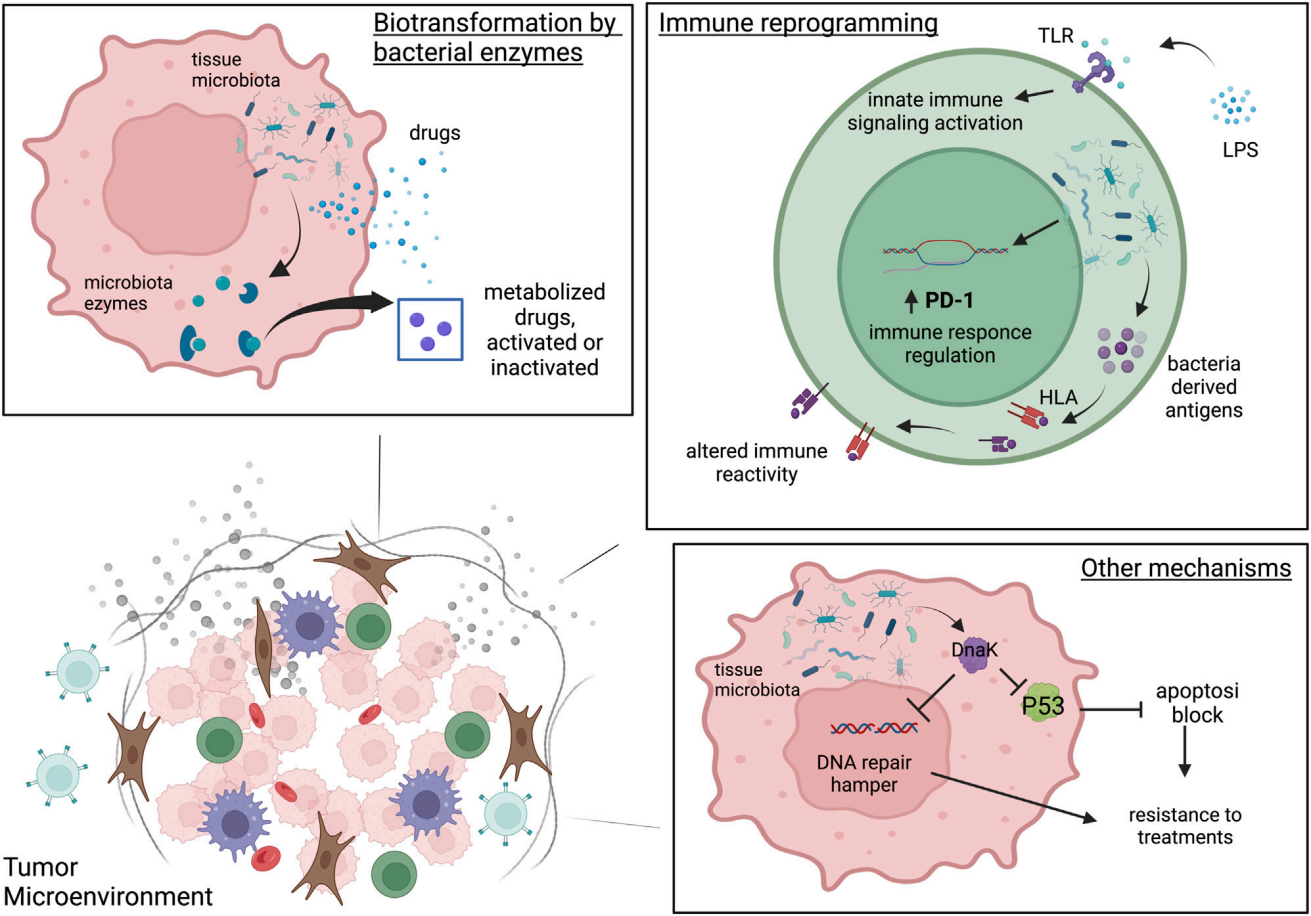
The three microbiota-mediated mechanisms of influence on cancer therapies. Biotransformation by bacterial enzymes. In tumor cell intratumoral bacteria can produce enzymes able to metabolize drugs and consequently activate or inactivate them. Immune reprogramming. Intratumoral bacteria can reprogram the TME immunity through the activation of Toll Like receptors and the innate immunity activation. Intratumoral microbiota can modulate PD-1 expression on CD4^+^ CD8+T cells and may affect T cell immune reactivity and immune function by introducing antigens derived from bacteria on tumor HLA-I and HLA-II molecules both on tumor cells and antigen presenting cells. Other mechanisms. In tumor cells the intratumoral bacteria can interfere with therapy response through the alteration of apoptosis, cell cycle and DNA repair by proteins of bacterial origin very similar to their human counterparts such as DnaK. Created with BioRender.com.

In addition to biotransformation and shaping of the immune microenvironment, the tissue microbiota can interfere with therapy response through other mechanisms, like altering apoptosis, cell cycle and DNA repair. These mechanisms may be dependent on proteins of bacterial origin, such as DnaKs. Indeed, it has been shown on SCID mice that *Mycoplasma fermentans* infection promotes lymphomagenesis through the action of a protein, DnaK, an HSP70 capable of interfering in various cellular mechanisms (Zella et al., 2018). In Zella’ study it was also shown *in vitro* that *M. fermentans* DnaK can reduce p53 activity by binding to USP10, one of the most important regulators of p53. This mechanism is capable of inducing resistance to treatments with 5-FU and Nutlin. DnaK is also able to hamper the PARP1 catalytic activity, an important actor in DNA repair mechanisms, and DNA-PKcs, required for non-homologous end joining in both dsDNA repair and V (D) J recombination. Of note, when the DnaK of *E. coli* were tested they observed that p53 activity was increased, the opposite effect observed by *M. fermentans* DnaK. The three mechanisms are represented in Figure 1. Of note that each species can adopt different strategies of interaction with the cell that can have clinical implications and potentially interfere with the response to therapies. For example, *Streptococcus gallolyticus* promotes human colon cancer cell proliferation only if in direct contact with the cell, increasing β-catenin, c-Myc and PCNA, which can be drug targets (Kumar et al., 2017). *Escherichia coli* produces the genotoxin colibactin, which promotes tumor growth in mice model of colon cancer by inducing senescence, which in turn can interfere with the response to therapies (Cougnoux et al., 2014).

## Cancer therapy shapes microbiota

Cancer creates a dysbiosis condition not only in the gut microbiota but also in tissue microbiota, and it is known that chemotherapy and immunotherapy are able to exacerbate the dysbiosis state, which potentially can worsen the therapy efficacy and lead to adverse events. The knowledge on the effects of the therapies on the tissue microbiota is scarce, contrary to what is known for the gut microbiota. However, an increase in intestinal permeability is often associated with a dysbiotic condition of the intestinal microbiota, which leads to an increase in the translocation of microbes to other sites. The administration of 5-fluorouracil (5-FU) to rats is able to perturb the oral and gut microbiota composition leading to an increase in Gram-negative anaerobes species and an increase in translocation of microbes to the mesenteric lymph nodes (Von Bültzingslöwen et al., 2003). Similar to 5-FU, CTX alters the composition of gut microbiota and leads to an increased intestinal permeability and to translocation of several Gram-positive species into mesenteric lymph nodes and spleen (Viaud et al., 2013). Therefore, it is highly probable that the tissue microbiota can also be modified by therapies.

## Therapeutic strategies for microbiota manipulation

Most of the therapeutic strategies that involve microbiota focus on the manipulation of the gut microbiota, which can be easily manipulated even through diet. However, new knowledge about tissue microbiota role in response to therapies makes it a considerable target of manipulation. Below, we report the latest innovations on the manipulation of the microbiota to improve the outcome of anti-cancer therapies, trying to keep the tissue microbiota as the focus (Table 1).

**TABLE 1:** Summary table of all the microbiota manipulation methods reported in the text.

Strategy	Tumor/model	Treatment	Effects	Citation
Antibiotics subministration	Pancreatic ductal adenocarcinoma mouse model	G418 + Gemcitabine	Antibiotic treatment enhance the anticancer activity of gemcitabine in mouse model	Geller et al. (2017)
	568 patients affected by Melanoma	Penicillin followed by cephalosporins and quinolones or vancomycin + immune checkpoint inhibitors (ICIs)	Penicillin + cephalosporins and quinolones affect responce to ICIs. Vancomycin has no effect on patients survival	Mohiuddin et al. (2021)
	Non-smal cell lung cancer	antibiotics + ICIs (meta-analysis)	Antibiotic use before or during treatment with ICI leads to a median OS decrease by more than 6 months	Lurienne et al. (2020)
	C57BL/6 mice model	CRISPR-Cas-3-encoding phages as selective antimicrobial for *Clostridium difficile*	Lytic activity and additive antimicrobial effect between lysis and bacterial genome degradation achieved	Selle et al. (2020)
Bacteria based cancer therapy	Lung adenocarcinoma mouse model	*E. coli* DH5α-lux/βG that express β-Glucoronidase +9ACG	Bacteria localize and replicate in human tumor xenografts. DH5α-lux/βG and 9ACG combination significantly delayed the growth of tumors	Cheng et al. (2008)
	3 patients with advanced and/or metastatic solid tumors considered sensitive to 5-FU	*S. typhimurium* (VNP20009) expressing the *E. coli* Cytosine Deaminase (CD) + 5-FU	Selective colonization in tumor tissue. Conversion of 5-FC to 5-FU.	Nemunaitis et al. (2003)
	Melanoma, breast cancer, colon cancer mouse model	*S. typhimurium* (VNP20009) expressing *Pseudomonas* sp carboxypeptidase G2 (CPG2) + different prodrugs	The CPG2-expressing bacteria alone reduce the growth of tumors. In the presence of prodrugs activated by CPG2, the oncolytic effect greatly increased	Friedlos et al. (2008)
	Bladder tumors rat model	Bifidobacterium infantis-mediated thymidine kinase (BI-TK) + ganciclovir (GCV) (BI-TK/GCV)	Bifidobacterium infantis-mediated TK/GCV suicide gene therapy system inhibit rat bladder tumor growth, possibly inducing apoptosis	Tang et al. (2009)
	Brain tumor rat model, canine spontaneous tumor, human leiomyosarcoma	*Clostridium* naviy-NT spores	Tumor size reduction	Roberts et al. (2014)
	Colon cancer mouse model	ΔppGpp *Salmonella* strain expressing cytosine deaminase	IL-1β and TNF-α were markedly increased in tumors colonized by ΔppGpp *Salmonellae*. The increase was associated with tumor regression	Kim J.E et al. (2015)
	Colon cancer mouse model	*S.Typhimurium* expressing cytolysin A (Cly A)	Significant suppression of both primary and metastatic tumors and prolonged survival in mice	Jiang et al. (2013)
	Colon cancer mouse model	*E. coli* strain K-12 expressing cytolysin A (ClyA) + radiotherapy	Significant tumor shrinkage and even complete disappearance of tumors. Inhibitory effect on lung metastasis	Jiang et al. (2010)
	Hepatocarcinoma mouse model	*S. typhimurium* engeniered with endostatin and Stat3-specific small interfering RNA	Decreased cell proliferation, induced cell apoptosis and inhibited angiogenesis. Combined treatment elicit immune system regulation by regulation of CD4+/CD8+ T cells and cytokines production	Jia et al. (2012)
Fecal microbiota transplantation	10 patients with anti–PD-1–refractory metastatic melanoma	6 combined tratment cycles of Anti-PD-1 therapy (nivolumab) + oral stool capsule (from donors that achieved complete response after anti PD-1 treatment)	Clinical responses in three patients, including two partial responses and one complete response	Baruch et al. (2021)
	16 melanoma patients refractory to anti–PD-1	15 patients received FMT + pembrolizumab	FMT transplant induced clinical benefit in 6 of 15 patients, and induced rapid and durable microbiota perturbation	Davar et al. (2021)
Probiotics	30 treatment-naive patients with metastatic renal cell carcinoma with clear cell and/or sarcomatoid histology and intermediate- or poor-risk disease	Nivolumab plus ipilimumab with or without daily CBM588 (bifidogenic live bacterial product)	PFS significantly longer in patients receiving nivolumab-ipilimumab with CBM588 than without. The response rate was higher in patients receiving CBM588 but not significant	Dizman et al. (2022)
	Colon cancer mouse model	The animals were treated with *Lactobacillus* acidophilus, and Bifidobacterium bifidum	miR-135b, miR-155, and KRAS were increased in the colon cancer mice group compared to the control group in both the plasma and the colon tissue samples. The probiotics consumption decreased their expression. Moreover, the miR-26b, miR-18a, APC, PU.1, and PTEN expressions were decreased in the colon cancer mice group compared to the control group and the consumption of the probiotics increased their expressions	Heydari et al. (2019)
	Melanoma murine model	germ free mice were first FMT using donor stool from a complete responder (CR) patient to anti–PD-1 blockade, followed by subministration of Bifidobacterium longum, or *Lactobacillus* rhamnosus GG or water + anti PD-1 therapy	Impaired antitumor response to anti–PD-L1 treatment and significantly larger tumor in mice receiving probiotics compared with control mice. Significantly reduced frequency of interferon-γ (IFN-γ) positive CD8^+^ T cells in tumors of probiotic-treated mice versus controls. A trend toward fewer IFN-g CD4^+^ T helper 1 (TH1) cells in tumors from mice receiving probiotics versus control	Spencer et al. (2021)
Prebiotics	Melanoma mouse model	Anti cancer treatment and high-fiber diet (FD)	Increased frequency and absolute numbers of total Dendritic Cells and cDC1s. Better spontaneous tumor growth control	Lam et al., 2021
Symbiotics	61 patients with advanced esophageal cancer	30 patients received symbiotic during neo-adjuvant chemotherapy, 31 patients no symbiotic treatment	Reduced occurrence of adverse events of chemotherapy through adjustments to the intestinal microbiota	Motoori et al. (2017)
	Intestinal mucositis rat model	*Lactobacillus* fermentum BR11 and fructo-oligosaccharide (FOS) + 5-fluorouracil (5-FU)	FOS do not confere therapeutic benefits in mucositis rats. L. fermentum BR11 has the potentially reduce inflammation of the upper small intestine	Smith et al. (2008)

### Bacterial depletion by antibiotics

The effect of the use of antibiotics on the efficacy of therapies is still controversial. The subministration of antibiotics is often used to prevent the onset of infections in patients undergoing chemotherapy or treatments capable of inducing immunosuppression. The treatment of cancer patients with antibiotics should worsen the response to therapies as it exacerbates the condition of dysbiosis, but in some cases enhance the response to treatment. As reported by Geller and others (2017), Ciprofloxacin enhances gemcitabine response. However, recent studies also underline the importance of the type of antibiotics used, the timing and the duration of the administration. A study by Mohiuddin et al. (2021) found that the response of patients to immune checkpoint inhibitors (ICIs) is negatively affected by the use of Penicillin followed by cephalosporins and quinolones, while vancomycin has no effect on the survival of patients. A meta-analysis by Lise Lurienne (2020) reports that antibiotic treatment in NSCLC before or during treatment with ICI leads to a median Overall Survival (OS) decreased by more than 6 months. Regarding the tissue microbiota, the intracellular localization protect bacteria from those antibiotics with limited or no cellular penetration ability (Imbuluzqueta et al., 2010; Abed and Couvreur, 2014), but not to treatments with cell penetrating doxycycline (Fu et al., 2022). This is certainly information to be taken into consideration for the definition of manipulation of *in situ* strategies. Surely, the use of antibiotics is important in case of infections but carefully selecting the type of antibiotic according to the target microorganisms can help a greater response to treatments. In order to prevent the side effects deriving from the use of antibiotics on the microbiota and on the patient, new products that can selectively affect taxa associated with disease have been developed. This new approach has been described in a recent study that proposes the use of CRISPR-Cas-3-encoding phages as selective antimicrobial for *Clostridium difficile* (Selle et al., 2020)*.* This type of approach is only at the beginning but could provide a good alternative solution to the use of broad range antibiotics.

### Bacteria based cancer therapy

Since 1920s, the medical field has been fascinated by the possibility of using bacteria-based cancer therapy. Since Corey’s toxin has been demonstrated to cure cancer, this hypothesis has been further explored. Today, it is known that many anaerobic bacteria have the ability to target and to kill tumor cells, such as *Salmonella*, *Clostridium*, *Listeria* and *Escherichia coli*. The application of these bacteria in the therapeutic field can be different. In fact, bacteria can be: used as vectors for their ability to target tumors; engineered for the production of pro-drug enzymes capable of activating drugs; used for the expression of controlled cytotoxic agents only in tumor cells; used to stimulate an immune response or to target the tumor stroma. Here are some examples of cancer therapy strategies that use engineered bacteria. Some drugs do not easily enter the cell and engineered bacteria can provide an excellent solution to introduce into the tumor cell all the necessary for the production of drugs. Glucuronide prodrugs may display selective anti-tumour activity against tumours that accumulate β-glucuronidase. Polar glucuronide prodrugs do not easily enter cells due to their charged carboxyl group. Cheng group (2008) generated an *E. coli* DH5α that express β-Glucoronidase gene cluster for the activation of prodrug 9ACG in 9AC and they also showed that the bacteria localize and replicate in human tumor xenografts and produce substantial antitumor activity in combination with systemic 9ACG prodrug therapy. Some bacteria produce prodrug-converting enzymes capable of optimizing the effectiveness of some drugs, one of these is Cytosine Deaminase (CD). From here the idea of using an attenuated strain of *S. typhimurium* (VNP20009) expressing the *E. coli* CD to be administered together with 5-FU. This method has been tested in a pilot study that confirmed an increased production of functional CD in the tumor and an increased conversion of 5-FC to 5-FU (Nemunaitis et al., 2003). The same vector has been used for the delivery of carboxypeptidase G2, a dimeric zinc dependent exopeptidase produced by Pseudomonas *sp. Strain RS-16* that has the ability to cleave the C-terminal glutamate moiety from folic acid and its analogues, which are a target molecule for chemotherapy. The delivery of carboxypeptidase G2 by *S. typhimurium* (VNP20009) vector showed an enhanced antitumor efficacy when administered in conjunction with prodrug (Friedlos et al., 2008). *Bifidobacterium infantis* has been used as vector to transport a prodrug enzyme of e *herpes simplex virus type I*, the thymidine kinase/ganciclovir (HSV1-TK/GCV), in a rat model showing that this target approach can inhibit the tumor growth by inducing apoptosis (Tang et al., 2009). Another strategy is to use modified bacteria to induce therapeutic benefits. *Clostridium* bacteria species have the ability to lyse tumor cells growing in hypoxic environments. Therefore, an attenuated strain of *Clostridium* novyi (C. novyi-NT) was produced and injected in a rat orthotopic brain tumor model, dogs with spontaneous canine tumors and a human patient with advanced leiomyosarcoma. In all cases, the treatment induced a tumor-localized response and tumor size reduction (Roberts et al., 2014) even if it does not eradicate all tumor cells. Perhaps its use in combination with other cancer treatments will give better results. A non-toxic strain of *Salmonella* have been used to treat CT26 tumor-bearing mice in order to examine bacteria-mediated immune responses and the results showed that the ΔppGpp *Salmonella* strain has the ability to activate inflammasome and activate several citochines confirming an antitumoral activity (Kim et al., 2015) Tumor-targeting bacteria can also be engineered to express cytotoxic agents with intrinsic antitumor activity such as cytolysin A (Cly A). Cly A is a pore forming hemolitic protein produced by *Paratyphi A* and also *E. coli* and *S.Typhimurium*. In 2 studies the expression of ClyA in *E. coli* or *S.Typhimurium* was controlled using inducible or constitutive promoters and in both cases an inhibition of the tumor was observed (Jiang et al., 2010; 2013). To target basilar mechanisms for cancer development, Jia and others (2012) cloned an attenuate *S.Typhimurium* with endostatin, an inhibitor of vessels generation, and a siRNA against stat3 in order to disrupt angiogenesis and inhibit proliferation. They tested the strain in orthotopically implanted hepatocarcinoma obtaining a downregulation of VEGF expression and an increase of cytokines expression and of CD4+/CD8+ T cells. The bacterial based cancer therapy can potentially offer as many opportunities for how many bacterial molecules interact with drugs, but at the moment most of the studies are still at the preclinical level.

### Fecal microbiota transplantation and probiotics

Above all, it is evident that the intestinal microbiota of those who respond to the therapies is different from that of non-responders, so the idea of modulating the microbiota in order to recreate the favorable conditions of the responders is an enormous opportunity. The strategies to restore the gut microbiota diversity are the fecal microbial transplantation (FMT) and the use of probiotics, prebiotics or synbiotics other than diet. These strategies are not specific for the tissue microbiota, but certainly indirectly, they can induce changes even at a distance, in the different niches but no studies are reporting this connection at the moment. FMT from healthy donors or responders to therapy has shown good success in immune checkpoint blockade (ICB)-resistant patients, increasing tumor immune infiltrate and increasing therapy-associated metabolites in serum (Baruch et al., 2021; Davar et al., 2021). Several clinical trials combining FMT with ICB (NCT03772899, NCT 04521075, NCT04924374, NCT04951583) are ongoing, but FMT is a very complex technique as well as the selection of donors. Therefore, some groups are experimenting with the possibility of transplanting only consortia of well-defined bacteria or the use of prebiotics and probiotics to induce a positive change in the microbiota. An example is the randomized phase 1 study (NCT03829111) where 30 treatment-naive patients with metastatic renal cell carcinoma have been treated with Nivolumab plus ipilimumab with or without CBM588, a bifidogenic live bacterial product containing *Clostridium butyricum.* The results suggest that the combination with CBM588 enhances the clinical outcome extending the Progression Free Survival (PFS), but larger studies are needed (Dizman et al., 2022). Probiotics are “live microorganisms which when administered in adequate amounts confer a health benefit on the host” (Sánchez et al., 2017). The administration of probiotic strains can protect the intestinal mucosa and consequently limit the translocation of microbiota to other sites. They can also reduce the side effects of anti-cancer therapy, prevent infections but also interfere with molecular mechanisms increasing the expression of tumor suppressor miRNAs and decreasing the level of the oncogenes, which can be advantageous for cancer treatment (Heydari et al., 2019). But there are also studies reporting a worse outcome when probiotics are administered in preclinical models and clinical cohorts treated with ICB (Spencer et al., 2021). Prebiotics are selectively fermentable, non-digestible oligosaccharides or ingredients that cause alterations in the composition and activity of gut microbiota. The subministration of prebiotics should promote the balance of bacteria in the colon and the production of their specific metabolites, which may have a valuable effect on anti-cancer treatments (Raman et al., 2013; Gibson et al., 2017). There are some evidence of an influence of prebiotics on ICB treatment in pre-clinical and clinical studies by acting in a positive modulation of T cells and reprogramming of tumor microenvironment (Lam et al., 2021). Symbiotics are a combination of probiotic bacteria and growth-promoting prebiotic ingredients that should selectively stimulate the increase and the activity of the probiotics. Symbiotic support to anticancer therapies has been little investigated but in general are administered to ameliorate the side effects due to therapies, even if their utility is still debated. In fact, while Motoori and others (2017) reported a decrease in the severity of diarrhea in esophageal cancer patients receiving neoadjuvant treatment, Smith and others (2008) did not report any amelioration in a rat model of intestinal mucositis. Although for pro-prebiotics and synbiotics there are not many studies that confirm their contribution as treatments that improve the response to therapies and side effects, their contribution cannot be excluded. With regard to these strategies, more extensive studies are needed and to be able to verify how much a fecal transplant or the use of pro/pre-biotics or synbiotics can affect the tumor microenvironment.

## Conclusion and future perspectives

The understanding of the many roles of microbiota in health and disease has improved in the last years revealing its involvement in fundamental mechanisms such as metabolism and immunity and suggesting the opportunity to use it as a marker and/or as a target to treat diseases. However, this knowledge is still at the beginning and mostly focused on the gut microbiota, in particular to the bacterioma. The profiling of the microbiota of specific niche such as the tumor microbiota is in its nascence, but it is already being understood that it should not be underestimated in terms of opportunities both for a holistic comprehension of the mechanisms that lead to tumor etiopathogenesis and for the improvement of therapies. The future of personalized medicine resides in holistic approaches that consider the tumor as part of a complex organism in which several factors contribute to the homeostasis of the system. The microbiota is taking part in these approaches, albeit only in research and not in clinical practice. In order to implement the clinical practice it is necessary to reveal the functional role of the microbiota in different contexts and in different locations but above all to create tools, technologies and approaches that allow its study. One of the next challenges is the development of *in vivo or ex vivo* models that allow the discrimination of the tissue or intestinal microbiota effects and the integration with meta-omics approches to evaluate how the system affects drug metabolization. Some good results have been achieved with *in vitro* systems such as HuMiX (Shah et al., 2016) and RapidAIM (Li et al., 2020), but the limit remains the systemic approach. Reproducing the conditions of the TME considering all the actors is a challenging objective, not only because the TME is made up of different cell types, but also because the microbiota is a complex combination of different microorganisms. In fact, as pointed out above, most of the studies concern the bacteriome, but the microbiota is also made up of viruses, fungi and protozoa. The microbial communities composing the microbiota interact with each other and an imbalance of one or the other can modify inter-kingdom interactions and compromise the health of the host (Pattaroni et al., 2022). An example above all is the occurrence of candidiasis due to the use of antibiotics. The mycobiome is the set of fungal communities that reside in our body and represents less than the 0.1% of total microbiota, but they represent a major cause of infectious morbidity and mortality in immune-compromised individuals (Nucci and Anaissie, 2001). As the bacterial microbiota, the fungal microbiota is present at different anatomical sites, and can colonize tissues as described by Aykut et al. (2019). Mycobiota is involved in inflammatory response in various diseases and their role is also emerging in the response to therapies, in fact, it can regulate the immunosuppressive microenvironment after radiotherapy (Shiao et al., 2021). Another important component of the microbiota which is poorly considered in the systemic point of view is the viroma. The existence of oncogenic viruses is well known, as is the association of some viruses with different types of tumor. Hence, some viruses establish persistent or latent infections in their hosts with subclinical effects, but which in the long run can contribute to the pathological background of the patient. Moreover, one of the largest components of the microbiota are bacteriophages. Some studies reported changes in the gut virome associated with desease onset and progression (Norman et al., 2015; Nakatsu et al., 2018; Clooney et al., 2019) and some preclinical studies have demonstrated the inflammatory potential and the interaction with the immune system (Zhang L. et al., 2018; Gogokhia et al., 2019; Sweere et al., 2019), but we are at an early stage in understanding the role of virome and its interaction with the other kingdoms. However, in the last two decades some groups started investigate the potential use of phage-based therapy to treat bacterial infections (Jennes et al., 2017; Schooley et al., 2017; Dedrick et al., 2019), in particular multiple-drugs resistant bacteria, but they are mostly case reports and larger studies are required**.** In conclusion, revealing the functional role of resident microbiota plus the interactions between the microbiota, cancer and cancer therapies will lead to improvement of current treatment efficacy and to the creation of personalized approaches that integrate the gut and the tumor resident microbiota profile.
